# Thinking about time: identifying prospective temporal illusions and their consequences

**DOI:** 10.1186/s41235-022-00368-8

**Published:** 2022-02-16

**Authors:** Brittany M. Tausen

**Affiliations:** Seattle Pacific University, 3307 3rd Ave West, Seattle, WA 98119 USA

**Keywords:** Prospective time judgments, Temporal illusions, Time perception, Judgment and decision making

## Abstract

Time is fundamentally abstract, making it difficult to conceptualize and vulnerable to mental distortions. Nine preregistered experiments identify temporal illusions that characterize prospective time judgments and corresponding consequences for decision making in a variety of domains. Using visual illusions as a grounding metaphor, studies 1–4 demonstrated that the temporal distance between two dates was perceived as closer together as those two dates were imagined further into the future (e.g., Vanishing Point); the length of a single day whether negative (e.g., a 12 h illness—Study 2a) or positive (e.g., 12 h with a good friend—Study 2b) was estimated to feel longer when embedded within a short versus long trip (e.g., the Delbouef Illusion); a 60 min activity was expected to go by more quickly when adjacent activities were 90 (vs. 30) min (e.g., Ebbinghaus Illusion); and a 9 + 1 day vacation was expected to be considerably lengthier than an 11–1 day vacation (e.g., Representational Momentum). Four additional studies explored moderating factors (Studies 5 and 6) and the impact of distortions on downstream non-time judgments including the forecasted emotional intensity of a negative event (Study 6), estimations of fair monetary compensation for lost time (Study 7), and willingness to make prosocial time commitments (Study 8). Implications for uncovering additional temporal illusions as well as practical applications for leveraging the relativity of prospective time to achieve desired cognitive and behavioral outcomes are discussed.

## Significance statement

The way that people perceive and think about time is both widely variable and highly consequential. Knowing how people systematically conceptualize time can provide insight into the emotions and decisions associated with an event, which may be particularly important for future time judgments given the utility of prospection to guide behavior. The current work: (a) identifies novel illusions that characterize the way people think about prospective time and (b) proposes practical applications for leveraging the relativity of prospective time judgments to achieve desired cognitive and behavioral outcomes. Beyond contributions to the time perception literature, the current work has practical applications for affective forecasting and decision making.

## Introduction

The way that people perceive and think about time is both widely variable and highly consequential. Indeed, a well-established body of research has demonstrated that time is subjectively constructed and susceptible to a menu of illusions (Bar-Haim et al., 2010; Eagleman, 2008; Maglio et al., 2013; Sackett et al., 2010). Individual and cultural differences (Bar-Haim et al., 2010; Chan & Saqib, 2021; de la Fuente et al., 2014; Hancock & Rausch, 2010; O'Brien et al., 2011), the valence and construal of an event (Droit-Volet & Gil, 2009; Hu & Maglio, 2018; Kanten, 2011; Wittmann & Paulus, 2008), the self-relevance of a period of time (Christian et al., 2012), and even one’s spatial orientation (Maglio & Polman, 2014), to name but a few, can impact subjective perceptions of, or estimations about, time. That such a broad array of factors can alter temporal perception emphasizes the complexity of understanding the causes and consequences of misconstruing time.

Much of what is known about time perception is grounded in episodic memory research (Draaisma, 2006; Munawar et al., 2018; Schroots et al., 2004; Wolf & Zimprich, 2020) or perceptual studies that ask participants to recreate the duration of different stimuli (Block & Gruber, 2014; Fountas et al., 2021; Zakay & Block, 1997). Both of these approaches are inherently connected to the ability to recall past events by mentally transcending the present (Haj et al., 2013). Mental Time Travel is not, however, constrained to the past (Suddendorf & Corballis, 2007). Rather, a burgeoning body of evidence explicates the human ability to prospect (Szpunar, 2010). This work has demonstrated that thinking about the future is not only common (Beaty et al., 2019; Christian et al., 2013), but also adaptive (Schacter et al., 2007; Schacter et al., 2012, 2017; Szpunar, 2010), underscoring the need to understand how people think about time that has yet to be experienced.

Despite much overlap in the way that past and future events are construed, there are also notable differences (see Beaty et al., 2019; Caruso, 2010; D’Argembeau & Demblon, 2012; Rubin, 2014; Schacter et al., 2012; Szpunar, 2010). Functional asymmetries and the hypotheticality of future-oriented thought, alone, provide some evidence to suggest that expectations, rather than experience, play a particularly prominent role in the construal of future temporal events (Beaty et al., 2019; Rasmussen & Berntsen, 2013; Spronken et al., 2016). Considering these differences and the utility of prospection, the current body of work explored how long hypothetical events are expected to feel. Specifically, the term ‘prospective time judgments’^1^ is utilized here to refer to conceptualizations of discrete and hypothetical units of time that could be experienced in the future (e.g., an hour-long dentist appointment next month).

### Consequences of misperceiving future time

A paucity of research has focused exclusively or explicitly on the temporal perception of discrete events in the future and those that do often look at the perceived temporal distance between “now” and target events (Caruso et al., 2013; Christian et al., 2012; Liberman et al., 2007; Zauberman et al., 2009). Such investigations have demonstrated that the perceived temporal distance to, and sense of personal connection with, future events have significant consequences for judgments and behaviors related to motivation and self-control (Hershfield, 2011; Kim & Kim, 2017; Macrae et al., 2014; Peetz et al., 2009; Rutchick et al., 2018). Research has also demonstrated systematic biases associated with the construal of future events, such as overestimating the duration of their emotional impact (van Dijk et al., 2008; Wilson & Gilbert, 2013; Wilson et al., 2000, but also see Levine et al., 2012) and underestimating their required completion time (Buechler et al., 1994). Not only are these prospective time judgments inaccurate, they are consequential. Developing this line of thought, it is important to catalogue conceptualizations of time that has yet to be experienced in order to determine how such perceptions might alter the cognitions and behaviors associated with mentally simulating the future.

### Factors that shape prospective time judgments

The current work focuses primarily on two promising, but under-researched, areas of prospective time judgments—the impact of context and expectations. As detailed above, the very nature of prospection is hypothetical, leaving people to rely more heavily on their expectations rather than their actual experiences. This over-reliance on expectations helps to explain the general positivity bias that colors the future (Rasmussen & Berntsen, 2013; Salgado & Berntsen, 2020) as well as the persistence of unrealistic time judgments that characterize the planning fallacy (Buechler et al., 1994). Individual differences such as optimism and rumination also impact the construal of the future (Beaty et al., 2019). Of course, expectations about future events extend beyond their anticipated emotion. Surprisingly little research, however, has explored other types of expectations about future events such as their anticipated duration (e.g., today is going to be a long day, our vacation is going to fly by)—a gap the current research aimed to address.

When considering the past, the context in which a remembered event is embedded can help to explain differing perceptions of time. For example, older adults perceive time to pass more quickly than younger adults, a phenomenon thought to be associated with the overall fraction of one’s life that is represented by a particular event (Landau et al., 2018). Relatedly, older adults group time into larger chunks (e.g., that happened in the 80s) whereas younger adults have smaller category boundaries (e.g., that happened when I was 8) for temporal events, which preserves the larger contextual backdrop for each individual event (Landau et al., 2018). Context, whether people view an event myopically or acknowledge surrounding events, also helps explain the impact bias as well as strategies to minimize its potency (Wilson et al., 2000). That estimates of temporal duration for specific events are not perceived in isolation, but rather influenced by the salience of surrounding events and the overall backdrop of one’s life suggests that prospective time judgments may also be sensitive to the nuance of adjacent events or the temporal boundaries within which they are situated.

### Conceptualizing the abstract

Time is undeniably abstract, making it difficult to conceptualize, communicate, and measure. In everyday life, people circumvent this difficulty by associating time with the more concrete medium of space. We speak of pasts that lay behind us, vacations that feel miles away, and unpleasant encounters that lurk around the corner. Such metaphors ground the concept in easily understood terms, and their use reveals how space is exploited to help people communicate to one another about time (Clark, 1973; Lakoff & Johnson, 1980). Likewise, research methodologies often utilize highly spatialized language (e.g., very short vs. very long; very near vs. very far) and measurement tools (e.g., physical gestures, timelines) to quantify conceptualizations of time (Caruso et al., 2013; Casasanto & Jasmin, 2012; Christian et al., 2012; Miles et al., 2011). Given the utility of space to think about time and that temporal and spatial perception are both characterized by illusions, perceptions of space were leveraged in the current work to help ground investigations of prospective time judgments.

### Current research

Five experiments identified distorted prospective time judgments using classic spatial illusions as a metaphorical guide. The Vanishing Point, famously illustrated by railroad tracks seeming to converge in the distance (Gillam, 1973, 1980); the Delboeuf and related Ebbinghaus Illusions (Coren & Girgus, 1978; Delboeuf, 1865), demonstrating an object’s size is biased by the size of a surrounding object(s); and Representational Momentum (Freyd & Finke, 1984; Hubbard, 2015), where people overestimate the size of objects that have been growing and underestimate the size of objects that have been shrinking, were all investigated. Four additional experiments investigated moderating factors (i.e., distance, similarity and speed) and whether prospective temporal illusions distorted consequential non-time judgments (i.e., emotional impact, fair monetary compensation, willingness to help).

A priori hypotheses, analyses, and sample sizes for each of the 9 studies were pre-registered prior to data collection at Aspredicted.org. On the basis of results from a pilot study a medium effect size, *d* = .50, was estimated. A power analysis indicated that a sample size of at least 86 participants per condition was required to have 90% power to detect such an effect. Pre-registered sample size was set to 100 participants per condition. The studies were reviewed and approved by Seattle Pacific University. Participants were recruited from Amazon’s Mechanical Turk for nominal pay.

## Experiment 1: vanishing point

**Fig. 1:**
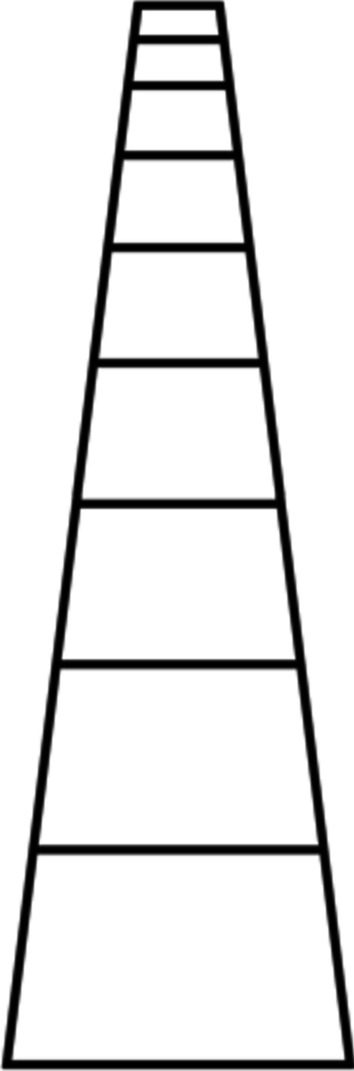
Two-dimensional visualization of the Vanishing Point Illusion where the horizontal lines begin to converge at a vanishing point in the distance

Often depicted by a railroad track going off into the distance, the Vanishing Point^2^ is one of the most widely familiar spatial distortions (Fig. 1). This illusion is also imbued with a variety of potential temporal analogues. Zauberman et al. (2009) detailed one of these, demonstrating that the subjective perception between now and incrementally increasing time points does not follow a linear function. These findings are consistent with the notion that the perception of time, like space, is warped by distance. Study 1 sought to corroborate this exploration in order to determine if the exact same amount of time (i.e., 1 month) in the future would be perceived as shorter than 1 month in the present. It was hypothesized that two dates 1 month apart in the distant future would feel closer together than two dates 1 month apart in the near future.

### Methods

Two hundred participants completed the study. The study employed a single factor (Temporal Distance: Now vs. In Six Months) between-participants design. Participants were asked to consider the span of one month of time as if it started today or as if it started 6 months from today. They indicated their perceptions of the two dates that bookended the month by rating how *far apart* and *bunched together* the dates felt, how *long of a line* represents the distance between the dates, and how *quickly* the time between the dates would pass, each on 100 point analogue scales.

Participants also rated how *busy* they would be and the *amount of stuff* that would happen during the month, also on 100 point analogue scales. These items served as control variables in order to isolate the perception of a month above and beyond differences in what people might do within that month.^3^

To ensure they understood the task, participants completed two manipulation checks: the start date (i.e., today vs. 6 months from today) and the length of time between the two dates that they were being asked to imagine (i.e., one month). Finally, all participants reported demographic information, were thanked, and debriefed.

### Results

Three participants were eliminated for incomplete responses and 37 participants for failing one or both of the manipulation checks. Data for the remaining 160 participants (86 Male, *M*_age_ = 36.21, *SD* = 11.56) were submitted to independent-samples *t*-tests.

**Fig. 2:**
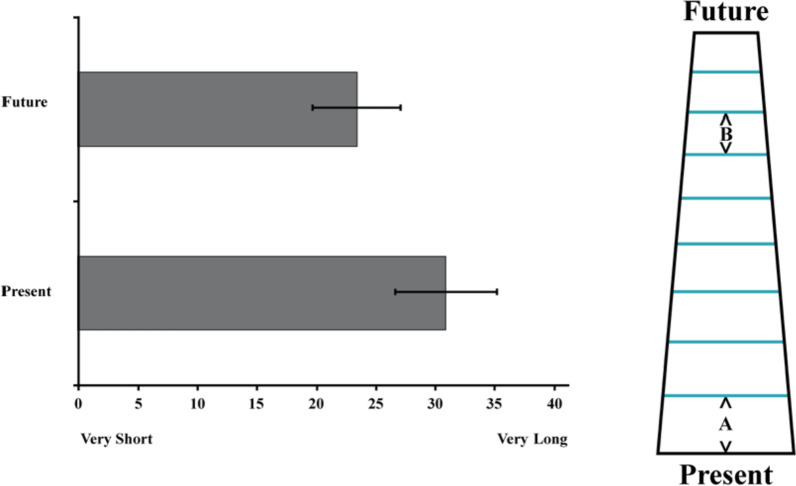
The left panel depicts the perceived length of 1 month as a function of temporal distance (now vs. 6 months from now). Error Bars represent 95% Confidence Intervals. The right panel illustrates the temporal analogue of the Vanishing Point Illusion in Study 1 by depicting the relative perceived size of 1 month now (**A**) and 6 months from now (**B**). Pixels representing target judgments (**A**, **B**) were calculated to reflect the relative proportions of the mean judgments for each condition

The dependent measures were collapsed into a composite ‘distance’ score (*α* = .92), with items recoded such that lower scores reflect a closer perceived distance. As hypothesized, the two dates that bookend a month were perceived as significantly closer together when that month was imagined in the far distance (*M* = 23.40, *SD* = 15.37) versus in the present (*M* = 30.93, *SD* = 20.95, *t*(158) = 2.62, *p* = .010, *d* = .40, 95% CI_difference_ [1.85, 13.20], see Fig. 2.

The control measures were also collapsed into a scale (*α* = .84), with lower scores reflecting less busyness. There was no difference in perceived busyness within the month, regardless of the month being far away (*M* = 57.58, *SD* = 25.39) versus in the present (*M* = 61.99, *SD* = 23.66), *t*(158) = 1.13, *p* = .260, 95% CI_difference_ [− 3.30, 12.12],^4^ and moreover, using this scale as a covariate in the analysis above does not change the effect, *F*(1, 157) = 6.05, *p* = .015, *η*_p_^2^ = .04.

### Discussion

Much like the Vanishing Point Illusion, Study 1 demonstrated a distortion in the perception of a fixed unit of time (i.e., 1 month) as a function of temporal distance. Put simply, one month felt different as a function of when in time it was located. Complimenting findings from Zauberman et al. (2009), the current work suggests it is not only the relationship between now and future time points, but also the explicit relationship between future time points that is compressed at a temporal distance. This is true even when controlling for potential differences in how busy one imagines being during a target time period. Taken together, these findings corroborate evidence suggesting that temporal distance distorts prospective time judgments.

## Experiment 2a: the Delboeuf Illusion

**Fig. 3:**
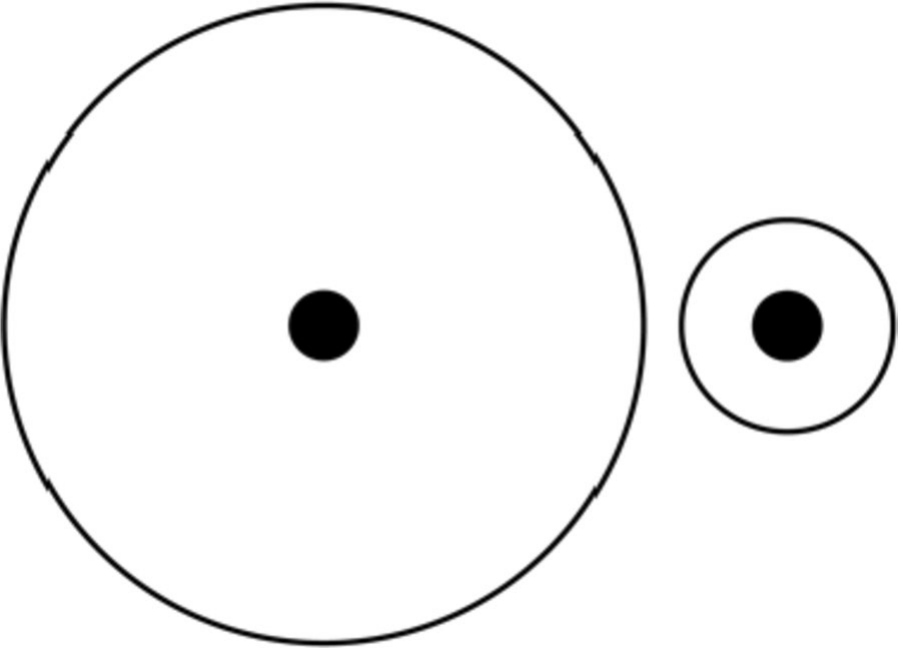
Visualization of the Delboeuf Illusion. Although the center circles are identical in size, the one on the right looks larger as a function of the smaller surrounding circle

The Delboeuf Illusion is a basic context illusion which demonstrates that two identical target circles are perceived to be different sizes as a function of the size of a surrounding circle (Fig. 3). This is often depicted by a cookie on a big versus a small plate. Here, we explored whether estimations of a fixed length of time are also sensitive to the context in which it is embedded. It was hypothesized that a fixed length of time (e.g., 12 h) would be expected to feel longer when contextualized within a short (3 day) rather than a longer (9 day) window of time.

### Methods

One hundred and two participants completed the study (50 Male, *M*_age_ = 38.00, *SD* = 11.67), which employed a single-factor (Length of Vacation: Short vs. Long) within-participants design.

A short questionnaire asked participants to entertain two similar, but distinct, scenarios in a randomized order. Participants were asked to imagine that they got sick for a day in the middle of a 3 day and, separately, a 9 day vacation. It was made clear that the sickness only lasted 12 h and that by the next day they were feeling good as new and enjoyed the remainder of the vacation.

Following the imagery of each scenario, participants were asked to respond to a single question. The question prompted participants to consider their 3 (c.f. 9) day vacation and to estimate how long the day they spent sick felt on a 100 point analogue scale with appropriate anchors (i.e., very short, very long).

Next, participants were given a forced choice question about the relative length of the sick day. Response options (presented in a randomized order) included (a) 1 sick day during a 3 day vacation felt longer, (b) 1 sick day during a 9 day vacation felt longer, or that (c) 1 sick day during a 3 day vacation feels the same as 1 sick day during a 9 day vacation. Finally, participants reported demographic information, were thanked, and debriefed.

### Results

**Fig. 4:**
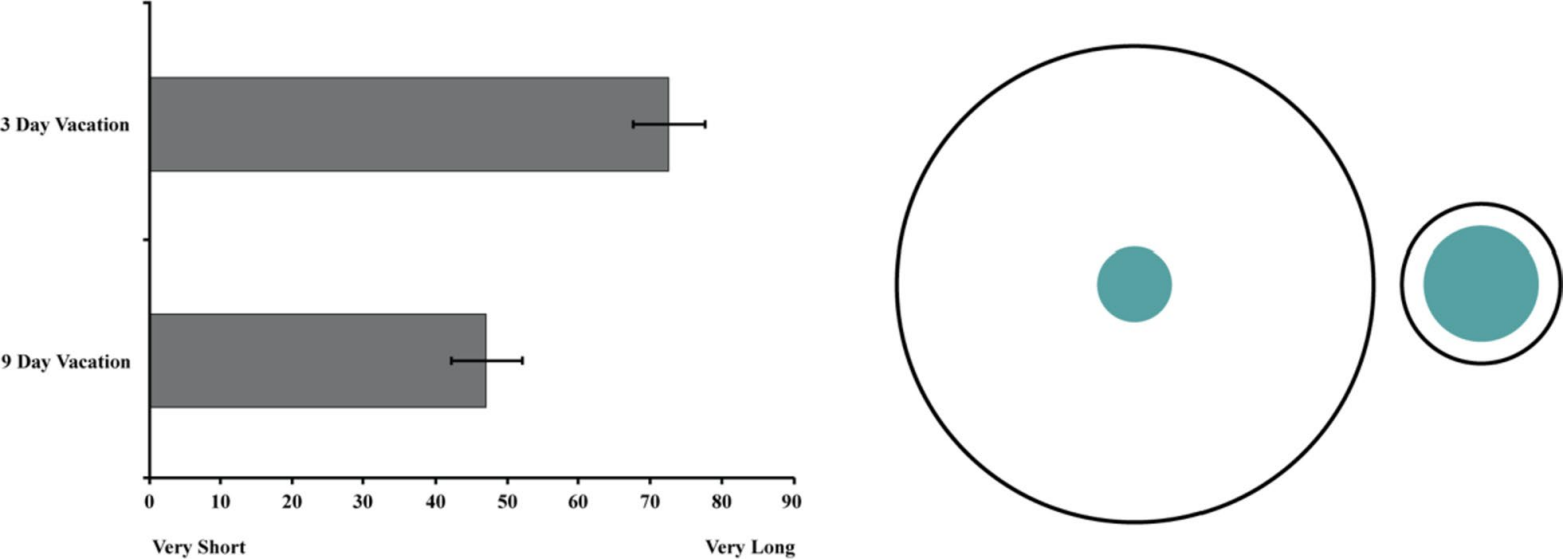
The left panel depicts the perceived length of 12 h spent sick as a function of vacation length. Error Bars represent 95% Confidence Intervals. The right panel illustrates the temporal analogue of the Delboeuf Illusion in Study 2a by depicting the relative perceived size of 12 h spent sick (green center circles) when embedded within a long (large black circle) versus short (small black circle) vacation. The number of pixels used to represent the diameters of the green target circles were calculated to preserve the relative proportions of the mean judgments for each condition

A paired samples *t*-test revealed a significant effect of Vacation Length *t*(101) = 8.05, *p* < .001, *d* = 1.01, 95% CI_difference_ [19.21, 31.77] such that a sick day felt shorter when contextualized within a long vacation (*M* = 47.06, *SD* = 25.01) compared to a short vacation (*M* = 72.55, *SD* = 25.41), see Fig. 4. Review of the distribution of forced choice responses revealed that the vast majority of participants (81.4%) perceived a sick day during a 3 day vacation to feel longer than a sick day during a 9 day vacation. A minority of participants (9.8%) thought a sick day during a 9 day vacation felt longer and 8.8% reported the sick days would feel the same in length. A Chi-square analysis of the forced choice responses (expected values set to 33.3%) confirmed that the statistical significance of this unequal distribution was not due to chance *χ*^2^(2) = 106.05, *p* < .001.

## Experiment 2b: the Delboeuf Illusion

Experiment 2a demonstrated that surrounding contextual factors shaped prospective time judgments when characterized by a particular valence pattern (e.g., a negative event disrupting a positive trip). To ensure that the illusion is not dependent upon valence, a replication of the study was conducted, reversing the valence pattern (e.g., a positive event in the middle of a negative trip). Specifically, participants imagined getting to spend an entire day with a good friend during a 3 (vs. 9) day work trip. All other details of the study were identical. If this prospective time illusion is not contingent upon valence, and in particular the frustration of losing positive time, a fixed length of time (e.g., 12 h) characterized by positive valence (e.g., seeing a good friend) should still feel longer when embedded within a short (3 day) rather than a longer (9 day) window of time.

### Methods

One hundred and one participants completed the study (59 Male, *M*_age_ = 41.25, *SD* = 11.00), which employed a single-factor (Length of Work Trip: Short vs. Long) within-participants design.

A short questionnaire asked participants to entertain two similar, but distinct, scenarios in a randomized order. Participants were asked to imagine that they got to spend a day with a friend in the middle of a 3 day and, separately, a 9 day work trip. It was made clear that they were only able to spend one day (12 h) with their friend during the trip and after their day with their friend they were back at work ‘bright and early’ the next day.

Following the imagery of each scenario, participants were asked to respond to a single question. The question prompted participants to consider their 3 (c.f. 9) day work trip and to estimate how long the day they spent with their friend felt on a 100 point analogue scale with appropriate anchors (i.e., very short, very long).

Next, participants were given a forced choice question about the relative length of the day with a friend. Response options (presented in a randomized order) included (a) 1 day with a friend during a 3 day work trip felt longer, (b) 1 day with a friend during a 9 day work trip felt longer, or that (c) 1 day with a friend during a 3 day work trip feels the same as 1 day with a friend during a 9 day work trip. Finally, participants reported demographic information, were thanked, and debriefed.

### Results

**Fig. 5:**
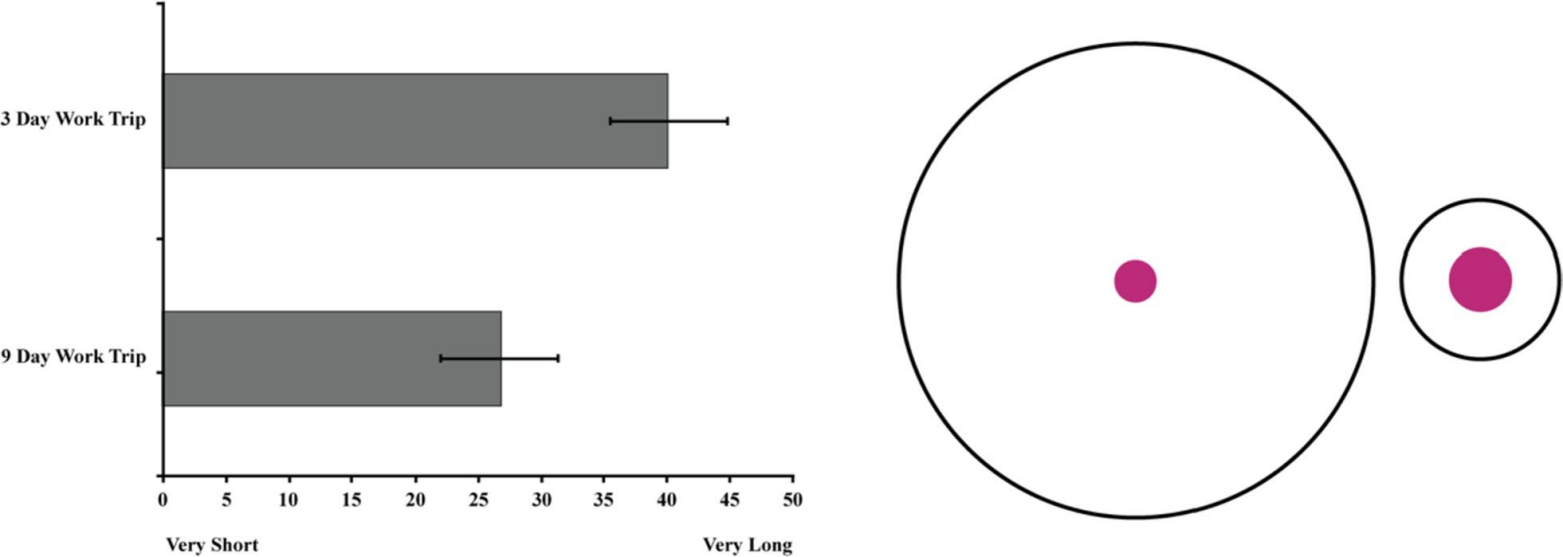
The left panel depicts the perceived length of 12 h spent with a friend as a function of work trip length. Error Bars represent 95% Confidence Intervals. The right panel illustrates the temporal analogue of the Delboeuf Illusion in Study 2b by depicting the relative perceived size of 12 h spent with a friend (pink center circles) when embedded within a long (large black circle) versus short (small black circle) work trip. The number of pixels used to represent the diameters of the pink target circles were calculated to preserve the relative proportions of the mean judgments for each condition

A paired samples *t*-test revealed a significant effect of Vacation Length *t*(100) = 5.31, *p* < .001, *d* = .56, 95% CI_difference_ [8.30, 18.21] such that a day with a friend felt shorter when contextualized within a long work trip (*M* = 26.78, *SD* = 23.21) compared to a short work trip (*M* = 40.04, *SD* = 24.06), see Fig. 5. Review of the distribution of forced choice responses revealed that the majority of participants (63.4%) perceived a day with a friend during a 3 day work trip to feel longer than a day with a friend during a 9 day work trip. A minority of participants (12.9%) thought 12 h with a friend during a 9 day work trip felt longer and 23.8% reported the days would feel the same in length. A Chi-square analysis of the forced choice responses (expected values set to 33.3%) confirmed that the statistical significance of this unequal distribution was not due to chance *χ*^2^(2) = 42.80, *p* < .001.

### Discussion

Like the Delboeuf Illusion, Studies 2a and 2b demonstrated that a fixed length of time is expected to feel different as a function of the size of the time window in which it is embedded. Further, these studies provide evidence that the illusion persists regardless of the valence of the estimated event. Of course, one might argue that the size of the vacation (work trip) itself (3 vs. 9 days) altered participants use of the time scale (which is inherently subjective) in each condition. Two primary counter points are worth considering. First, the majority of participants in both studies reported that a 1 day would feel longer during a 3 (vs. 9) day trip on the forced choice question lending additional credibility to the temporal illusion. A striking feature of spatial illusions is that even when knowing two objects are identical in size, a side-by-side comparison still gives rise to the perception that one is larger than the other. This phenomenon holds true for the current temporal illusion, minimizing concerns that the findings are driven solely by a differential use of the scale (see also Study 7 for downstream consequences of the Delboeuf Illusion using an objective non-time judgment). Additionally, an altered use of the scale would have been reflected in the proportions of the time judgments. For example, losing 1 day in a 9 day vacation results in losing approximately 11% of one’s trip whereas losing 1 day in a 3 day vacation results in losing approximately 33% of one’s trip. However, participants’ estimates of how long the day felt were vast overestimations of the percentage of trip lost (47.1% and 72.6%, respectively) if they were simply adjusting the scale to represent the duration of the entire trip. Together, these considerations point to a robust temporal illusion that cannot be explained simply by anchoring one’s judgments to a different scale.

Beyond context, these two studies also provide some insight into how valence impacts prospective time judgments. Comparing effect sizes of Study 2a and 2b suggests that this illusion of context (i.e., trip length) was stronger when the target time was negative (i.e., sick day) rather than positive (i.e., day with a friend). Of course, this comparison should be considered with caution as it is unclear if the magnitude of valence is equally matched across events (e.g., losing a vacation day might be considered more negative than seeing a friend is considered positive). Future work could probe this finding to determine the extent to which the temporal analogue of the Delbouef Illusion might be moderated by valence. If elements of time perception extend to temporal prospection (e.g., positive time goes by more quickly than negative time), then prospective time judgments should evidence both the valence illusion in addition to the contextual one. Indeed, the temporal illusions investigated here seem to act in tandem with (not eliminate) other documented misperceptions of time.

The current findings demonstrate that a fixed length of time is perceived differently as a function of the context in which it is embedded. These results are in line with recent work that has shown abstraction can alter time-related judgments (Hu & Maglio, 2018; Kanten, 2011). In a particularly relevant test of this hypothesis, Kanten (2011) showed that estimates of a task’s duration increased at a temporal distance. It is possible that these findings are related to multiple temporal illusions operating in tandem. To the extent that distant future (and thus more highly abstracted) windows of time are perceived as smaller (the Vanishing Point; Study 1), a fixed task might loom large within the smaller context (the Delbouef Illusion; Study 2).^5^ Future work, however, would be necessary to understand why abstraction would constrict a general time window without also constricting the target task. Independent of construal, the effects in Studies 2a and 2b provide direct empirical evidence that the size of a temporal frame distorts perceptions of a specified amount of time—within a narrow temporal frame a fixed unit of time is magnified whereas a wide temporal frame gives the illusion that the same amount of time is brief. Study 3 sought to extend the current findings by exploring the impact of surrounding temporal events rather than an encapsulating time frame.

## Experiment 3: the Ebbinghaus Illusion

**Fig. 6:**
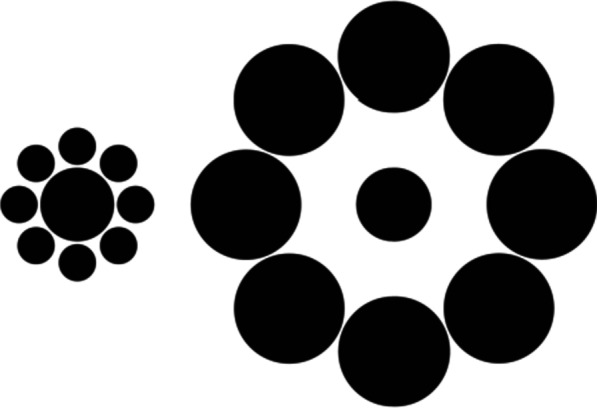
Visualization of the Ebbinghaus Illusion. Although the center circles are identical in size, the one on the left looks larger as a function of the smaller surrounding circles

Closely related to the Delboeuf Illusion, the Ebbinghaus Illusion (see Fig. 6) is also context-dependent. Unlike the Delboeuf Illusion, however, it is not one encompassing object, but several surrounding objects that give rise to the illusion. Two target circles identical in size appear different as a function of if they are surrounded by larger or smaller circles. When smaller circles flank the target circle, it appears significantly larger than when flanked by larger circles. Guided by this spatial metaphor, it was hypothesized that a fixed length of time (e.g., 60 min) should feel longer when surrounded by shorter (30 min) rather than a longer (90 min) events.

### Methods

One hundred participants completed the study (68 Male, *M*_age_ = 34.01, *SD* = 10.28), which employed a single-factor (Length of Surrounding Meetings: Short vs. Long) within-participants design.

A short questionnaire asked participants to entertain two similar, but distinct, scenarios in a randomized order. Participants were asked to imagine that they had a job where they see multiple clients back-to-back throughout the day. Participants were then shown their schedule depicting meetings with 5 separate clients. In the Short condition, meetings 1, 2, 4, and 5 were all 30 min whereas meeting 3 was 60 min. In the Long condition, meetings 1, 2, 4, and 5 were all 90 min whereas meeting 3 was 60 min.

Following the imagery of each scenario, participants were asked to respond to a single question. The question prompted participants to consider their 60 min meeting with Client 3 and to estimate how long the meeting felt on a 100 point analogue scale with appropriate anchors (i.e., very short, very long).

Next, participants were given a forced choice question about the relative length of their meeting with Client 3. Response options (presented in a randomized order) included (a) a 60 min meeting surrounded by 30 min meetings feels longer, (b) a 60 min meetings surrounded by 90 min meetings feels longer, or that (c) a 60 min meeting feels equally long regardless of the length of surrounding meetings. Finally, participants reported demographic information, were thanked, and debriefed.

### Results

**Fig. 7:**
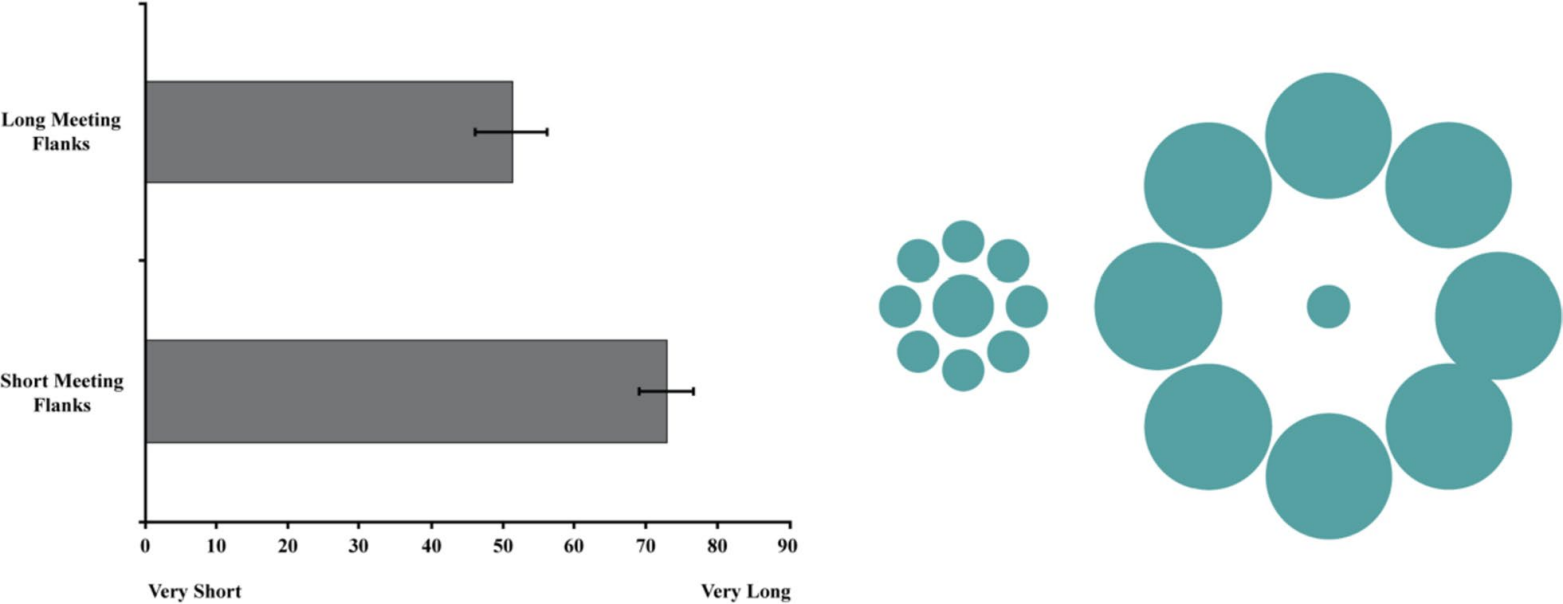
The left panel depicts the perceived length of a 60 min meeting as a function of surrounding meeting length. Error Bars represent 95% Confidence Intervals. The right panel illustrates the temporal analogue of the Ebbinghaus Illusion in Study 3 by depicting the relative perceived size of 60 min (green center circles) when surrounded by 30 (small circle flanks) versus 90 (large circle flanks) minute meetings. The number of pixels used to represent the diameters of the center green circles were calculated to preserve the relative proportions of the mean judgments for each condition

A paired samples *t*-test revealed a significant effect of Surrounding Meeting Length *t*(99) = 6.93, *p* < .001, *d* = .96, 95% CI_difference_ [15.40, 27.76] such that a 60 min meeting felt longer when surrounded by 30 min meetings (*M* = 72.67, *SD* = 19.22) compared to 90 min meetings (*M* = 51.09, *SD* = 25.30), see Fig. 7. Review of the distribution of forced choice responses revealed that the majority of participants (53%) perceived a 60 min meeting surrounded by 30 min meetings to feel longer than a 60 min meeting surrounded by 90 min meetings. A minority of participants (10%) thought a 60 min meeting felt longer when surrounded by 90 min meetings and 37% reported the meetings would feel the same in length. A Chi-square analysis of the forced choice responses (expected values set to 33.3%) confirmed that the statistical significance of this unequal distribution was not due to chance *χ*^2^(2) = 28.34, *p* < .001.

### Discussion

Study 3 demonstrated that a fixed unit of time (i.e., a 60 min meeting) was perceived differently as a function of the surrounding units of time (i.e., 90 vs. 30 min meetings). Like Study 2, this illusion was present even when directly comparing the two scenarios such that the majority of participants reported that a 60 min meeting would feel longer when surrounded by 30 min meetings than 90 min meetings. These findings corroborate and extend the findings from Studies 2a and 2b, suggesting that surrounding events influence the perceived duration of a fixed unit of time. The temporal analogue of the Ebbinghaus Illusion identified here also lends further credence to the notion that prospective temporal illusions are not dependent upon the valence of the target and contextual events. While Studies 2a and 2b isolated perceptions of a negative event within a positive frame and a positive event within a negative frame, Study 3 held constant the valence such that the target event and the surrounding events were all identical (meetings with clients). While the valence that characterizes time undoubtedly has the potential to impact perceptions of how quickly it passes, surrounding events, at least when making prospective judgments, also shape judgments about time.

## Experiment 4: representational momentum

Moving beyond context distortions, Study 4 sought to determine if perceptions of time are sensitive to expectations. Both spatial and temporal judgments are biased by the motion of the perceiver and the space/time being perceived (Miles et al., 2010). Being oriented toward a relevant location gives the illusion of closer proximity than does being oriented away from a location (Maglio & Polman, 2014) and future events (thought to be moving toward us) are perceived to be closer than past events (thought to be moving away; Caruso et al., 2013). Such illusions of momentum extend beyond spatial and temporal judgments and are thus not unique to egocentric perceptions. For example, events are perceived to be more likely if their probability is presumed to be increasing rather than decreasing (Maglio & Polman, 2016) and a line that appears to be growing on a screen will be estimated as longer than a line that appears to be shrinking even when the final lines are identical in length. These illusions exist because the direction of movement is extrapolated. Temporal events may also appear to have grown or shrunk relative to initial expectations about their duration. As such, it was hypothesized that a fixed amount of time (10 days) would feel longer if time has been added (i.e., 9 days + 1 day) than if time has been taken away (i.e., 11 days–1 day).

### Methods

One hundred and two participants (62 Male, *M*_age_ = 37.51, *SD* = 12.55) completed the study, which employed a single-factor (Condition: Control, Increased Day, Decreased Day) within-participants design.

A short questionnaire asked participants to imagine three distinct, but similar scenarios in a random order. All participants imagined that they had gone on a vacation to a place that they had always wanted to visit. In the Control condition, participants were told they had booked a 10 day vacation and were later notified that their return flights might be altered, but in the end they remained unchanged. In the Increased Day condition, participants were told they had booked a 9 day vacation and were later notified that their flights had been rescheduled to leave a day later, making their vacation 10 days in total. In the Decreased Day condition, participants were told they had booked an 11 day vacation and were later informed that their flights had been rescheduled to leave a day earlier, making their vacation 10 days in total.

Following the mental simulation of each scenario, participants were asked to consider their 10 day vacation and to estimate the perceived length of their vacation on a 100 point analogue scale with appropriate anchors (i.e., very short, very long). Finally, all participants reported demographic information, were thanked, and debriefed.

### Results

**Fig. 8:**
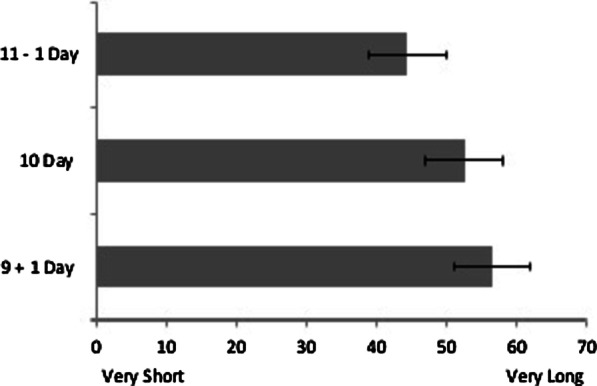
Perceived length of a 10-day vacation as a function of condition. Error Bars represent 95% Confidence Intervals

A repeated measures ANOVA revealed a significant effect of condition on estimates of vacation length *F*(2, 202) = 27.68, *p* < .001, *η*_p_^2^ = .22 (see Fig. 8). Post hoc pairwise comparisons (Bonferroni corrected) revealed a significant difference between all conditions such that the 10 day vacation in the Increased Day condition (*M* = 56.44, *SD* = 27.93) was perceived to be significantly longer than in the Decreased Day condition (*M* = 44.26, *SD* = 28.19), *t*(101) = 6.56, *p* < .001, *d* = .43, 95% CI_difference_ [8.49, 15.86] and the Control condition (*M* = 52.55, *SD* = 28.14), *t*(101) = 2.53, *p* = .026, *d* = .14, 95% CI_difference_ [.84, 6.94]. The Decreased Day condition was also perceived to be significantly shorter than the Control condition, *t*(101) = 5.17, *p* < .001, *d* = .29, 95% CI_difference_ [5.10, 11.47].

### Discussion

Study 4 demonstrated that temporal momentum (whether time increased or decreased relative to initial expectations) impacts perceptions of a fixed unit of time (i.e., 10 days) resulting in a temporal illusion analogous to representational momentum. While previous work has demonstrated that the locus (past vs. future) and perceived temporal proximity of temporal events are shaped by egocentrically relevant approach/avoid behaviors (Caruso et al., 2013; Maglio & Polman, 2014; Miles et al., 2010), the current study uniquely demonstrates a misperception of time as it pertains to momentum relative to expectations about an event’s duration rather than its motion toward or away from the perceiver. It could be argued that the mechanism driving this temporal illusion was a basic context effect (10 days is more than 9, but less than 11) or a differential sensitivity to change as a function of the initial length of the trip (i.e., Weber’s Law). Notably, however, if participants were employing this strategy, losing 1/11th of a trip should result in a smaller difference between the day lost and control condition whereas gaining 1/9th of a trip should result in a larger difference between the day gained and control condition. Comparing the means for each condition to the control, however, provided evidence to the contrary, such that participants’ time estimates were more sensitive to the day lost condition than the day gained condition. These findings corroborate findings that losses often loom larger than gains (Kahneman & Tversky, 1979; McGraw et al., 2010, but also see Gal & Rucker, 2018) and suggest that valence and expectations may both be at play in this temporal illusion.

## Experiment 5: moderating the Ebbinghaus Illusion

Studies 1–4 demonstrated a wide variety of prospective time illusions. Studies 5–8 explored factors that moderate the established temporal illusions as well consequences associated with distorted perceptions of time. Extending the spatial metaphor for temporal illusions employed here, many contextual factors attenuate the strength of visual illusions. For example, the Ebbinghaus Illusion is known to weaken as a function of two key factors: distance between the center circle and the surrounding circles and incongruence between the shape of the target object and the surrounding objects. The greater the distance between surrounding circles and the target circles, the more similar the two center circles appear (Jaeger & Grasso, 1993). Additionally, if the surrounding objects are shaped differently (i.e., triangles instead of circles), the illusion begins to dissipate (Coren & Miller, 1974). Experiment 5 sought to examine similar moderating factors by increasing the temporal distance between the target time period and the comparison time periods as well as changing the ‘shape’ or nature of the comparison periods. If similar factors moderate spatial and temporal illusions, the temporal analogue of the Ebbinghaus Illusion should be weakened by these manipulations.

### Methods

Three hundred and two participants (153 Male, *M*_age_ = 36.21, *SD* = 11.81) completed the study. The study employed a 2 (Flank: Long vs. Short) × 3 (Condition: Control, Temporally Distant, Different Activity) mixed-design with repeated measures on the first factor. Each participant was exposed to both the Long and Short Flank conditions. Participants were randomly assigned to either the Control, Temporally Distant, or Different Activity condition.

A short questionnaire asked participants to entertain two similar, but distinct, scenarios in a randomized order. All participants were asked to imagine that their favorite television program played on Wednesday evenings for 60 min. In the Short Flank conditions, participants were asked to imagine that the other weeknights (MTRF) the show was 30 min. In the Long Flank condition, participants imagined the show was 90 min MTRF. The days of the week along with the length of the tv program were then displayed for participants in a table.

Participants in the Control condition saw the two scenarios as described above. Participants in the Temporally Distant condition were told that the show only aired once a month (e.g., 30 min Jan, 30 min February, 60 min March, 30 min April, 30 min May) and participants in the Different Activity condition were told that they exercised on Monday, Tuesday, Thursday, and Friday nights, but watched their favorite tv program on Wednesdays.

Participants then answered the following question, “Please consider the 60 min you get to watch your favorite show on Wednesday nights and estimate how long the show would feel to you.” Participants responded using a 100 point analogue scale with appropriate anchors (i.e., very short, very long). Finally, all participants reported demographic information, were thanked, and debriefed.

If a temporal analogue of the Ebbinghaus illusion exists, we should replicate the results of Study 3—an activity with a set time (e.g., 60 min) would feel shorter if surrounded by longer activities (90 min) than if it were surrounded by shorter activities (30 min). If similar factors moderate spatial and temporal distortions, the strength of the illusion should be minimized in the Temporally Distant and Different Activity conditions.

### Results

**Fig. 9:**
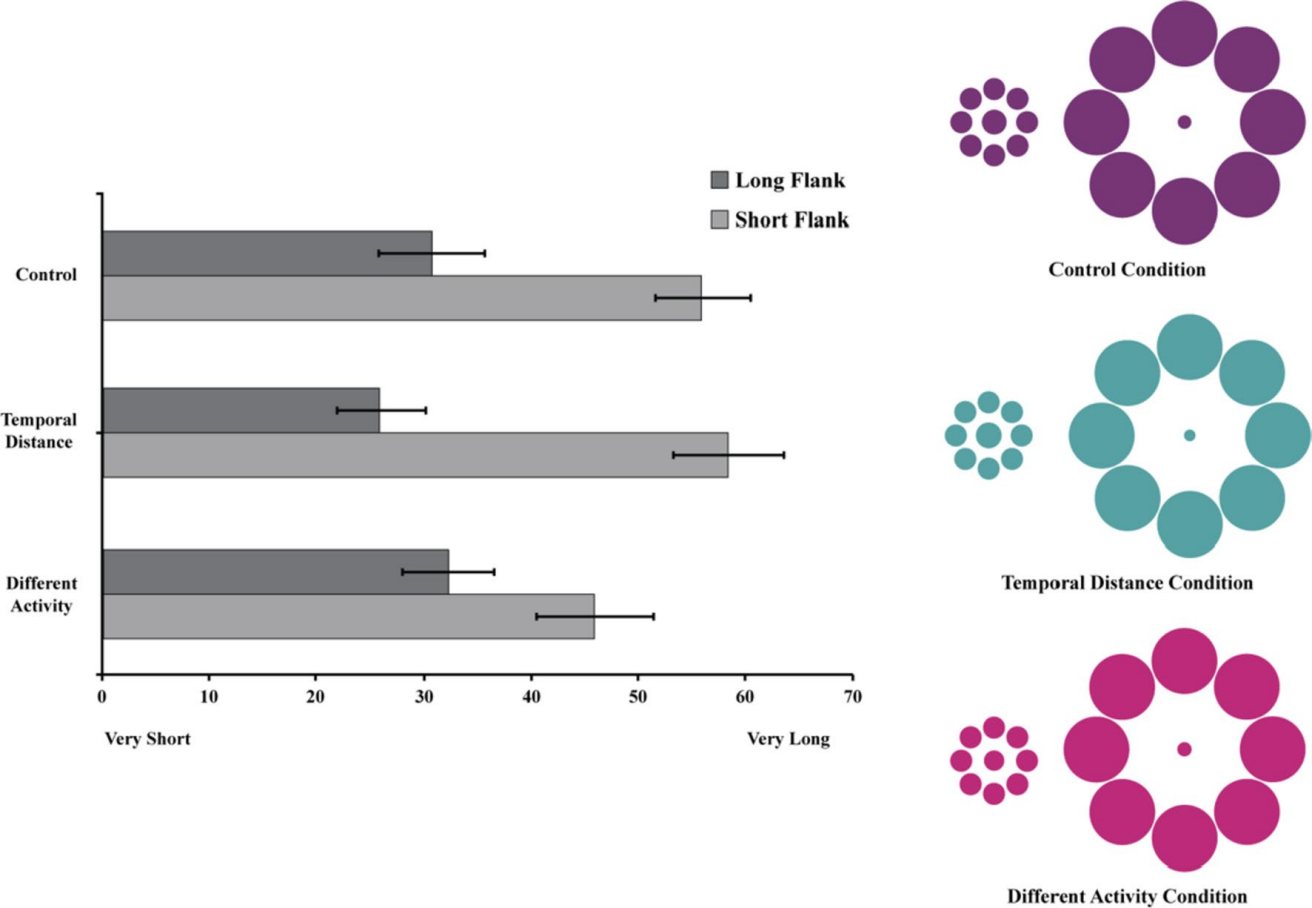
The left panel depicts the perceived length of a 60 min television program as a function of Surrounding Activity Length (Short Flank: 30 min vs. Long Flank: 90 min) and moderator condition. Error Bars represent 95% Confidence Intervals. The right panel illustrates the temporal analogues of the Ebbinghaus Illusion for each of the 3 moderating conditions in Study 5 by depicting the relative perceived size of 60 min (center circles) when surrounded by 30 (small circle flanks) versus 90 (large circle flanks) minute activities. The number of pixels used to represent the diameters of the center target circles were calculated to preserve the relative proportions of the mean judgments for each condition.

A mixed-model ANOVA revealed a significant main effect of Flank (Short vs. Long) on the perceived length of the target activity, *F*(1, 299) = 212.99, *p* < .001, *η*_p_^2^ = .42. Condition did not have a significant effect on the perceived length of the target activity, *F*(2, 299) = 1.28, *p* = .279, *η*_p_^2^ = .008. These effects were qualified by a significant Condition × Flank interaction effect, *F*(2, 118) = 11.10, *p* < .001, *η*_p_^2^ = .07 (see Fig. 9).

Post-hoc *t*-tests Bonferroni corrected revealed a significant difference in perceived size for all three conditions. An hour long tv show was perceived as longer when surrounded by 30 min viewings (*M* = 56.02, *SD* = 27.47) than 90 min viewings (*M* = 30.70, *SD* = 21.61), *t*(101) = 8.40, *p* < .001, *d* = 1.03, 95% CI_difference_ [19.35, 31.30]. An hour show in March was perceived as longer when the show was 30 min on adjacent months (*M* = 58.50, *SD* = 26.28) compared to 90 min (*M* = 25.97, *SD* = 20.74), *t*(99) = 10.65, *p* < .001, *d* = 1.37, 95% CI_difference_ [26.47, 38.59]. Finally, a 60 min show was perceived as longer when surrounded by 30 min of working out (*M* = 45.99, *SD* = 22.70) compared to 90 min of working out (*M* = 32.23, *SD* = 25.26), *t*(99) = 5.80, *p* < .001, *d* = .57, 95% CI_difference_ [9.06, 18.47].

To further explore the interaction effect, difference scores (perceived size of 60 min with 30 min flanks—perceived size of 60 min with 90 min flanks) were computed to quantify the magnitude of the temporal illusion. Planned comparisons revealed that the distortion in time perception did not differ between the Control (*M* = 25.32, *SD* = 30.43) and Temporally Distant condition (*M* = 32.53, *SD* = 30.55), *t*(200) =  − 1.68 *p* = .095, 95% CI_difference_ [ − 15.67, 1.25]. However, the illusion was significantly weaker in the Different Activity condition (*M* = 13.76, *SD* = 23.71) compared to both the Control *t*(200) = 3.01, *p* = .003, 95% CI_difference_ [3.98, 19.14] and the Temporally Distant conditions *t*(186.52) = 4.85, *p* < .001, 95% CI_difference_ [11.14, 26.40].

### Discussion

In addition to replicating the findings of Study 3, the present study demonstrated a reduction in the strength of this prospective temporal distortion when altering the similarity between the target time period and the surrounding time periods. Much like an incongruence in shape reduces the strength of the Ebbinghaus Illusion (Coren & Miller, 1974), an incongruence in activity type reduces the strength of the temporal illusion. The temporally distant condition, however, did not weaken the illusion. It is possible that this moderator did not impact time judgments because a competing illusion is at work when trying to conceptualize temporal distances to future events (see Study 1). While further research will be necessary to determine if greater temporal distances (e.g., 1 year between events) are substantial enough to abate the illusion, the compression of increasing temporal distances from now (Zauberman et al., 2009) may negate extended amounts of time between a target event and surrounding events. If this is the case, then prospective time judgments may be more resilient to the increased distance of surrounding objects than spatial judgments. Study 6 sought to probe another potential moderator of prospective temporal illusions inspired by representational momentum—speed.

## Experiment 6: the speed of time lost and emotional impact

Extrapolating the motion of objects in space requires knowledge of direction and speed. How quickly an object travels alters estimations of where it will be located at a later point in time. If a line appears to be shrinking very quickly (vs. slowly), estimations of the final line length will incorporate the speed. Faster shrinking will result in estimations of shorter lines relative to slower shrinking. From a temporal perspective, certain circumstances can make it seem like time is lost very quickly or slowly. Namely these perceptions are grounded in expectations (e.g., to say something happened quickly often implies that it happened sooner than expected). Thus, one way to make it appear that something happened quickly versus slowly, might be to alter expectations about when we should ‘expect’ something to happen. Prospective time judgments can then be made when the event is believed to occur prior to (or after) the set expectation. If speed similarly moderates spatial and temporal illusions, a fixed amount of time (30 years) should feel shorter if time was lost quickly (e.g., sooner than expected) rather than if time was lost slowly (e.g., later than expected).

### Methods

One hundred and five participants (44 Male, 1 non-binary, *M*_age_ = 36.15, *SD* = 12.29) completed the study, which employed a single-factor (Speed of Time Lost: Quickly vs. Slowly) within-participants design.

A short questionnaire asked participants to imagine two distinct, but similar scenarios in a counterbalanced order. In the Time Lost Quickly condition, participants were told, “Imagine that you have been married to your partner for 30 years. One day you get a phone call informing you that your partner had a heart attack and passed away very suddenly.” In the Time Lost Slowly condition, participants were told, “Imagine that you have been married to your partner for 29 years. One day you get a phone call informing you that your partner had a heart attack. Your partner survived the heart attack, but their health slowly declined over the next year. After 30 years of marriage, your partner passed away.” Following the mental simulation of each scenario, participants were asked to consider the 30 years they spent with their partner and to estimate how long those 30 years felt on a 100 point analogue scale with appropriate anchors (i.e., very short, very long). Next, participants were asked to directly compare the two conditions on perceived length and, separately, emotional impact using 100 point analogue scales. Larger numbers represented that time felt shorter / that the experience felt worse in the condition where a loved one was lost suddenly. The midpoint of the scale indicated that the time and emotion felt equivalent regardless of how suddenly the loved one was lost and smaller numbers suggested losing a loved one slowly made time together feel shorter / that the experience felt worse. Finally, all participants reported demographic information, were thanked, and debriefed.

### Results

A paired samples *t*-test revealed a significant effect of how quickly time was lost *t*(104) = 4.62, *p* < .001, *d* = .34, 95% CI_difference_ [6.13, 15.36] such that 30 years of marriage after losing a partner suddenly felt significantly shorter (*M* = 43.61, *SD* = 32.42) than 30 years of marriage after losing a partner slowly (*M* = 54.35, *SD* = 30.27). One-sample *t*-tests compared participants’ ratings of the perceived length of time and the perceived emotional impact to the midpoint of the scale (50). Numbers above 50 indicated that 30 years after a sudden loss felt shorter / worse whereas numbers lower than 50 indicated that 30 years after a slow loss felt shorter / worse. Results revealed that ratings of both the perceived length of time (*M* = 66.77, *SD* = 26.34) and perceived emotional impact (*M* = 63.14, *SD* = 28.48) significantly differed from the midpoint of the scale suggesting that after a sudden death 30 years felt shorter *t*(104) = 6.53, *p* < .001 and more negative *t*(104) = 4.73, *p* < .001 than 30 years of marriage that ended with a slow decline. A one-tailed Pearson’s Correlation revealed that estimates of time and emotional intensity were positively correlated *r*(105) = .16, *p* = .052, but failed to reach significance. Future research will be necessary to probe whether the trend seen here is spurious or represents a reliable connection between perceptions of how quickly time is lost and emotional intensity that holds across participants.

### Discussion

Study 6 demonstrates that the speed of time lost impacts perceptions of temporal duration. Aside from providing evidence to suggest perceived speed can impact the estimated length of a fixed time period, this study also demonstrated a consequential downstream judgment. Specifically, observation of the mean of the emotional impact item revealed that participants imagined a loss to have a more negative emotional impact when time was lost quickly than when time was lost slowly. While many factors likely contribute to the valence estimates (e.g., counterfactual thinking, quality of time imagined in final year together), this work highlights the complexity of the relationship between valence and temporal construal. While there is a complex relationship between valence and time estimations, here we show that short time can be perceived as negative. The remaining studies sought to explore other downstream consequences of temporal illusions.

## Experiment 7: Delbouef Illusion and monetary compensation

The common adage, ‘time is money’ suggests that the perceived length of an event might be associated with estimations of appropriate monetary compensation. If a set amount of time appears shorter as a function of being contextualized within a larger (vs. smaller) time frame (as Studies 2a and 2b suggest), then the temporal analogue of the Delbouef Illusion might result in systematically different estimations of fair compensation for the exact same amount of time. If downstream monetary consequences are associated with temporal distortions, participants should expect more compensation for a fixed length of time (e.g., 6 h) within a short (2 day) rather than a longer (4 day) window of time.

### Methods

One hundred and five participants completed the study. The study employed a single-factor (Length of Vacation: Short vs. Long) within-participants design.

A short questionnaire asked participants to entertain two similar, but distinct, scenarios in a randomized order. Participants were asked to imagine that they were going on a 2 (vs. 4) day vacation and that upon arriving at the airport they are informed that their flight had been delayed 6 h. Further, participants were told that to apologize for the inconvenience the airline offered to refund part of their $300 ticket.

Following the imagery of each scenario, participants were asked to respond to a single question. The question prompted participants to consider what amount of compensation would be fair for the delay. Responses were collected on a 100 point analogue scale with appropriate anchors (i.e., $0, $300).

Next, participants were given a forced choice question about the relative length of the delay. Response options (presented in a randomized order) included a) a 6 h delay during a 2 day vacation feels longer, b) a 6 h delay during a 4 day vacation feels longer, or c) a 6 h delay feels equally long regardless of vacation length. Finally, participants answered a manipulation check question about the differences between the two scenarios, reported demographic information, were thanked, and debriefed.

### Results

Fourteen participants failed the manipulation check and were eliminated from the study. Data analysis was performed on the remaining 91 participants (50 Male, *M*_age_ = 33.84, *SD* = 9.45). A paired samples *t*-test revealed a significant effect of Vacation Length *t*(90) = 3.58, *p* = .001, *d* = .24, 95% CI_difference_ [2.93, 10.24] such that participants expected more compensation when a 6 h delay affected a 2 day vacation (*M* = 68.20, *SD* = 27.06) compared to a 4 day vacation (*M* = 61.62, *SD* = 27.38). Review of the distribution of forced choice responses revealed that the majority of participants (57.1%) perceived a 6 h delay during a 2 day vacation to feel longer than a 6 h delay during a 4 day vacation. No participants reported that a 6 h delay during a 4 day vacation would feel longer. A Chi-square analysis of the forced choice responses (expected values set to 33.3%) confirmed the statistical significance of this unequal distribution was not due to chance *χ*^2^(2) = 48.29, *p* < .001.

### Discussion

The present study brings to light tangible consequences of the temporal analogue to the Delboeuf Illusion. Participants estimated fair monetary compensation for a 6 h flight delay to be significantly higher when taking a 2 day rather than a 4 day vacation. Adding to the downstream emotional consequences of temporal illusions (Study 6), these data suggest that the relationship between time and money is also susceptible to the effects of temporal distortions. This study also provides evidence that the temporal analogue of the Delboeuf Illusion (Studies 2a and 2b) persists when using an objective scale and considering more similar surrounding time frames (i.e., 2 vs. 4 days compared to the 3 vs. 9 days in Studies 2a and 2b).

## Experiment 8: Ebbinghaus Illusion and willingness to help

Operating on the assumption that if a time commitment is short an individual will be more willing to help, the final study investigated whether temporal illusions could be leveraged to nudge prosocial time commitments. Specifically, we explored individuals’ willingness to volunteer for a local charity. If the temporal analogue of the Ebbinghaus Illusion impacts downstream judgments, simply flanking a 2 h volunteer time slot with longer (i.e., 3 h) as opposed to shorter (i.e., 1 h) time slots should make individuals more willing to volunteer 2 h of their time.

### Methods

One hundred and two participants completed the study. The study employed a single-factor (Surrounding Timeslot Length: Short vs. Long) within-participants design.

A short questionnaire asked participants to entertain two similar, but distinct, scenarios in a randomized order. Participants were asked to imagine that they were approached by a friend who works at a local charity and asked to consider volunteering for an upcoming event. Participants were shown a list of available timeslots for the day that contained four 3 h (vs. 1 h) timeslots and a single 2 h timeslot. The order of the timeslots were presented such that the 2 h timeslot appeared in the middle of the day, flanked by two earlier and two later time slots of the appropriate length.

Following the imagery of each scenario, participants were asked to respond to a single question. The question prompted participants to consider the 2 h timeslot and to estimate how willing they would be to volunteer for the slot on a 100 point analogue scale with appropriate anchors (i.e., not at all willing, very willing). If the temporal analogue of the Ebbinghaus Illusion has downstream consequences for prosocial time commitments, participants should be more willing to help when a 2 h timeslot is surrounded by 3 h (vs. 1 h) time slots.

Next, participants were given a forced choice question about the relative length of the 2 h volunteer time slot. Response options (presented in a randomized order) included (a) 2 h feels longer when the other timeslots are 1 h, (b) 2 h feels longer when the other timeslots are 3 h, or (c) 2 h feels equally long regardless of the length of the other timeslots. Finally, participants answered a manipulation check question about the difference between the two scenarios, reported demographic information, were thanked, and debriefed.

### Results

Twenty-three participants failed the manipulation check and were eliminated from the study. Data analysis was performed on the remaining 79 participants (42 Male, *M*_age_ = 36.95, *SD* = 12.03). A paired samples *t*-test revealed a significant effect of Surrounding Timeslot Length *t*(78) = 6.63, *p* < .001, *d* = .65, 95% CI_difference_ [13.92, 25.88] such that participants were more willing to volunteer for a 2 h timeslot surrounded by 3 h timeslots (*M* = 71.57, *SD* = 27.86) compared to 1 h timeslots (*M* = 51.67, *SD* = 33.13). Review of the distribution of forced choice responses revealed that the majority of participants (57%) perceived a 2 h timeslot to feel longer when surrounded by 1 h timeslots. The remainder of participants reported that a 2 h timeslot felt longer when surrounded by 3 h timeslots (10.1%) or that a 2 h timeslot felt equally long regardless of surrounding timeslots (32.9%). A Chi-square analysis of the forced choice responses (expected values set to 33.3%) confirmed the statistical significance of this unequal distribution was not due to chance *χ*^2^(2) = 26.00, *p* < .001.

### Discussion

This final study demonstrated yet another downstream consequence of temporal illusions. Participants reported being significantly more willing to help when a 2 h volunteer slot was flanked by 3 h (vs. 1 h) time slots, suggesting that it may be possible to leverage temporal illusions not only to alter the impact of emotional experiences and the associated costs, but also to motivate individuals to act more prosocially. These data also provide corroborating evidence for the temporal analogue of the Ebbinghaus Illusion (Studies 3 and 5) utilizing longer surrounding time flanks (i.e., 1/3 h vs. 30/90 min).

## General discussion

Studies 1–4 identified distortions in how people think about future points in time. The experimental conditions giving rise to temporal misperceptions varied widely spanning temporal distance from now (Study 1), surrounding events (Studies 2a, 2b, and 3), and momentum (Study 4). Studies 5–8 then probed moderators of these illusions (i.e., similarity of temporal events, Study 5 and perceived speed of time lost, Study 6) and demonstrated implications for non-time judgments related to the anticipated emotional intensity of a negative event (Study 6), estimations of fair monetary compensation (Study 7), and willingness to help (Study 8).

Although identifying the theoretical underpinnings of prospective temporal illusions was beyond the scope of the current manuscript, the distortions identified here contribute to an impressive body of research identifying the malleability of time perception (Eagleman, 2008; Maglio et al., 2013; Sackett et al., 2010). Specifically, the current work identified a host of contextual factors have the potential to impact prospective time judgments—a form of time perception that has received surprisingly little direct attention. Contextual factors can be readily altered when mentally simulating the future and, while speculative, are likely to hold even as the scales change (e.g., minutes, years). As such, knowing how context influences prospective time judgments may be particularly useful to guide strategies to counteract (Kruger & Evans, 2004) or tactically leverage (Morewedge et al., 2013) temporal distortions, ultimately refining the utility of prospection.

### Practical implications

The distortions in prospective time judgments demonstrated here gave rise to practical consequences in myriad domains (forecasted emotion, monetary compensation, willingness to help). While acknowledging the widespread implications of these specific findings, we suspect that the utility of temporal misperceptions is much farther reaching. Perceiving the time between two future dates as negligible may exacerbate the amount of stress we experience when considering, for instance, a job interview and a cross-country move that fall a month apart on next year’s calendar or give rise to inflated estimations of how long a distant future task might take (Kanten, 2011). Moreover, the perceived impact of hedonic events may be dwarfed by situating them in broader timeframes (e.g., “You only have to spend an hour at the dentist *this year*”). A related flanking mechanism might be utilized to nudge actual time commitments or strategically guide schedule arrangements to elongate (or minimize) a central event. Finally, the way that activities end may prove critical. A long lecture may seem to fly when students get out 15 min early, whereas a short class may drag on forever if it runs 15 min late. Indeed, past research suggests that expectations about likelihood of events can alter their estimated hedonic impact (Buechel et al., 2017). Continuing to unpack these issues would afford valuable insight into when to combat versus exploit the observed illusions, as well as many others that might be inspired by the existing literature on time perception.

### Space and time

In much the same way that space is often utilized to help people conceptualize the abstract concept of time (Casasanto & Boroditsky, 2008; de la Fuente et al., 2014; Kanten, 2011; Maglio & Polman, 2014; Miles et al., 2010, 2011; Núñez & Sweetser, 2006), visual illusions were used here as a metaphor to ground investigations of prospective temporal illusions. Notably, however, the metaphorical connection between spatial and temporal illusions is limited. For example, there are important methodological differences between the experience of visual illusions and the mental simulation of prospective temporal illusions. Thus, visual illusions might be more closely related to distortions in experienced rather than anticipated time. Although temporal and spatial perceptions are related (Bueti & Walsh, 2009; Parkinson et al., 2014), the current findings do not address a causal link between spatial and temporal illusions. While it is possible that some of the same factors shape both temporal and spatial perceptions (e.g., context, motion), a number of differences between spatial perception and time perception also exist. In the current work, there was evidence that some temporal distortions may be more robust than analogous spatial distortions. Study 5, for example, demonstrated that increased distance between a target event and future events did not weaken the temporal illusion whereas the visual Ebbinghaus Illusion weakens in intensity when surrounding contextual shapes appear at greater distances from the target shape (Jaeger & Grasso, 1993). An interesting area for future research might be to more thoroughly probe the potential overlap between temporal and spatial distortions. Notably, these explorations could be mutually informative such that these two bodies of work could help to advance one another, rather than simply using spatial metaphors to help conceptualize time.

### Conclusion

Given widespread implications for planning and decision-making, the current body of work aimed to catalogue distortions and consequences of prospective time judgments. Future research will be necessary to determine whether the same distortions extend to real-time and retrospective time judgments. Such investigations will help determine whether future-oriented thought is uniquely vulnerable to context illusions or if common mechanisms distort how time is anticipated, experienced, and remembered.

## Data Availability

The datasets used and/or analyzed during the current study are available from the corresponding author on reasonable request.

## Footnotes

1 The term prospective time has also been used in reference to a paradigm utilized for time interval estimation judgments where participants are warned that they will need to recreate (or estimate) the duration of a particular stimulus before it is experienced (Haj et al., 2013; Taatgen et al., 2007).

2 The Vanishing Point Illusion is often illustrated in concert with the Ponzo Illusion and thus the two are easily confused. By itself, the Vanishing Point Illusion simply demonstrates how distance influences size perception (e.g., objects appear smaller with increased distance to a point where they ‘vanish’). The Ponzo Illusion demonstrates that estimates of the size of a particular object is distorted when depicted at different distances in a two-dimensional drawing. Take, for example, two lines of identical length placed over the top of a railroad track drawing. The line that is presented ‘closer’ where the railroad tracks look farther apart appears smaller than the line that is presented ‘farther’ where the railroad tracks look closer together. In essence, the Ponzo Illusion demonstrates how size perception is relative to other contextual cues. If the lines are really the same length, the one farther away should look smaller, if it does not, it is ‘perceived’ as being exceptionally large to reconcile having the same retinal projection as the line that is ‘up close’.

3 Previous work has established that the amount of episodic content associated with a given period of time decreases with increased temporal distance from now (D' Argembeau & Van der Linden, 2004; Spreng & Brian, 2006). Measurements of busyness were included to control for this phenomenon to ensure that any differences in time perception were independent of episodic content.

4 It is worth noting that the current study did not reveal differences in perceptions of how busy one would be during the present month as opposed to a month in the future. At face value, this finding is inconsistent with previous work which has shown less episodic content associated with future points in time (D' Argembeau & Van der Linden, 2004; Spreng & Brian, 2006) as well as perceptions that one will have more spare time in the future (Zauberman & Lynch, 2005). It is likely that these disparate findings stem from at least two key methodological differences. First, the dependent variables in question vary substantially across studies. It is possible that people perceive more ‘spare time’ in the future (Zauberman & Lynch, 2005), not because they are less busy or have fewer tasks to perform, but because they expect to be more efficient with their time. According to the planning fallacy, people regularly underestimate the amount of time future tasks will take. In addition, individuals likely hold intentions to be less wasteful with their time in the future (e.g., I won’t spend 2+ hours a day on social media next month). Second, previous work has often focused on smaller time units (a day rather than a span of 30 days) and has explicitly asked participants to identify specific events that will be occurring on those given days at different points in the future. In the current study, we assessed a more basic intuition about how busy a current vs. a future month would be. Given that episodic content has been shown to decline even one day in the future, it is perhaps unsurprising that when comparing the next 30 days to a 30 day period 6 months from now, meaningful differences do not emerge.

5 This would also be consistent with the Ponzo Illusion which demonstrates that lines of equal length appear different in size when they are overlaid on a two-dimensional drawing that depicts varying distances.
